# A Novel Method for Intelligibility Assessment of Nonlinearly Processed Speech in Spaces Characterized by Long Reverberation Times

**DOI:** 10.3390/s22041641

**Published:** 2022-02-19

**Authors:** Adam Kurowski, Jozef Kotus, Piotr Odya, Bozena Kostek

**Affiliations:** 1 Department of Multimedia Systems, Faculty of Electronics, Telecommunications and Informatics, Gdansk University of Technology, 11/12 Narutowicza Street, 80-233 Gdansk, Poland; adakurow@pg.edu.pl (A.K.); jozkotus@pg.edu.pl (J.K.); pioodya@pg.edu.pl (P.O.); 2 Audio Acoustics Laboratory, Faculty of Electronics, Telecommunications and Informatics, Gdansk University of Technology, 11/12 Narutowicza Street, 80-233 Gdansk, Poland

**Keywords:** acoustic sensing, speech transmission index (STI), speech intelligibility, acoustic quality

## Abstract

Objective assessment of speech intelligibility is a complex task that requires taking into account a number of factors such as different perception of each speech sub-bands by the human hearing sense or different physical properties of each frequency band of a speech signal. Currently, the state-of-the-art method used for assessing the quality of speech transmission is the speech transmission index (STI). It is a standardized way of objectively measuring the quality of, e.g., an acoustical adaptation of conference rooms or public address systems. The wide use of this measure and implementation of this method on numerous measurement devices make STI a popular choice when the speech-related quality of rooms has to be estimated. However, the STI measure has a significant drawback which excludes it from some particular use cases. For instance, if one would like to enhance speech intelligibility by employing a nonlinear digital processing algorithm, the STI method is not suitable to measure the impact of such an algorithm, as it requires that the measurement signal should not be altered in a nonlinear way. Consequently, if a nonlinear speech enhancing algorithm has to be tested, the STI—a standard way of estimating speech transmission cannot be used. In this work, we would like to propose a method based on the STI method but modified in such a way that it makes it possible to employ it for the estimation of the performance of the nonlinear speech intelligibility enhancement method. The proposed approach is based upon a broadband comparison of cumulated energy of the transmitted envelope modulation and the received modulation, so we called it broadband STI (bSTI). Its credibility with regard to signals altered by the environment or nonlinear speech changed by a DSP algorithm is checked by performing a comparative analysis of ten selected impulse responses for which a baseline value of STI was known.

## 1. Introduction

Objective measurements of the real world—on the one hand—require appropriate technology and—on the other hand—promote and accelerate development, sharing new principles, testing, and technology improvement, so it fits into a circle of innovation in technology as defined by Phillips [1]. It brings about new technology and, at the same time, new ways of using technology and services, and based on the feedback between both reinforces innovation. An example of the practical use of such a paradigm is the development of objective speech intelligibility measures in the domain of measurements in architectural acoustics, public address system design, occupant warning systems as well as sound systems for emergency purposes. Speech transmission index (STI) and its newer derivative measures such as rapid STI (RASTI), STIPA for public address, ETSI for telephone networks [2], or STITEL for telecommunication systems [3,4], along with devices, allow for validating compliance with audibility and intelligibility standardized requirements. All measures mentioned above are intended for objective measurement of speech intelligibility (SI) carried out as an automated process that does not require a laborious and costly procedure of performing subjective listening tests. However, it should be remembered that the best judge of speech intelligibility is the human ear. Therefore, there are many attempts to find a correlation between objective measurement results and subjective evaluation [5,6,7,8,9]. In general, the STI value can be determined using two ways, i.e., the direct method based on modulated signals or the indirect method based on the impulse response, according to IEC 60268-16 standard [10]. Using an STI-measuring meter, which is often a compact, hand-held device, is a very compelling way of obtaining fast and repeatable measurements associated with speech intelligibility. Moreover, STI or STIPA and other measures have a substantial drawback which disqualifies their usage in the case of, e.g., evaluation of the performance of algorithms designated for improvement of speech intelligibility of demanding conditions that require non-standard methods of acoustic treatment [11]. This concerns especially locations such as multi-story parking lots or train stations, which are strongly affected by environmental conditions influencing the acoustics of such sites. In the works of Gomez-Agustina et al. [12], Tronchin [13], and Yang and Moon [14], it was shown that air parameters, i.e., temperature or relative humidity, might significantly influence measures such as reverberation time (RT), STI score, or even subjective feeling of being annoyed by the environmental noise. Therefore, in some cases, a more sophisticated algorithm has to be applied to dynamically improve acoustic conditions in such spaces. An example of such an algorithm is provided in the earlier authors’ paper [11]. It is based on slowing down the speech rate, which improves subjectively assessed speech intelligibility. Unfortunately, such an operation is a nonlinear transformation applied to the speech signal. Consequently, it is not possible to use STI to measure the effect of such an algorithm on speech intelligibility, as standards defining the STI and STI-derived measures are designated to measure signals that are processed in a linear-only manner [10]. Hence, the STI measure does not apply to evaluating a nonlinear audio-processing algorithm. To mitigate this problem, we propose a modification of the STI measure to use it in the context of nonlinear operations altering speech for increasing its intelligibility.

Therefore, the aim of this paper is to propose a modified STI measure and check its credibility with regard to signals altered by the environment or nonlinear speech changed by a DSP algorithm. To achieve this goal, we introduce a notion of a broadband STI, called bSTI, derived from comparing the cumulated energy of the transmitted envelope modulation and the received modulation. To assess the validity of the proposed measure, we carried out a comparative analysis of ten selected impulse responses for which a baseline value of STI was known. They were measured in three types of spaces:

- A main utility space of a shopping mall (3 impulse responses with STIs of 0.35, 0.41, and 0.62),
- An above-ground parking hall (2 impulse responses with STIs of 0.37 and 0.58),
- A staircase of an office building (5 impulse responses with STIs of 0.33, 0.35, 0.39, 0.45, and 0.51).

The repeatability of the bSTI-based measurements and Pearson’s correlation with the STI measure are further investigated.

The paper is organized as follows. First, methods related to both speech intelligibility evaluation and speech quality are recalled. Then, the limitations of the STI measures are briefly discussed. They constitute the background of our research study. In Section 2, our method is introduced. This is followed by showing calculated correlation results. Finally, conclusions are derived, and future method development is presented. 

### 1.1. State-of-the-Art of the Speech Intelligibility Assessment Methods

Speech is featured by redundancy in many ways: acoustic, phonetic, and lexical. However, noise, distortions, interfering sounds, or reverberation negatively affects speech intelligibility or acoustic measures related to linguistically contrasting units [9]. As a result, speech may be audible but not intelligible. The issues related to the influence of various factors on speech intelligibility are discussed in detail by Assmann and Summerfield [15]. Among other observations, the authors address the amplitude-time dependence of the speech signal, which they refer to as “temporal envelope modulations”, i.e., reverberation disrupts natural variations in signal amplitude by filling in sections of silence and pauses. Analysis of the modulation spectrum provides a means of assessing the influence that various disturbances have on speech intelligibility. This approach has become the basis for developing an objective measure of speech intelligibility: the speech transmission index (STI) [16,17,18], as well as the articulation index (AI), introduced much earlier [19,20,21].

It should, however, be remembered that STI, introduced originally by Houtgast and Steeneken [16], addresses both background noise and reverberation [16,17]. Overall, it is said that there are three methods underlying speech intelligibility evaluation, i.e., Speech Intelligibility Index (SII), Speech Transmission Index (STI), and Articulation Index (AI) [22]. A thorough review of the follow-up of predicting speech intelligibility was brought by Ma et al. [3], who pointed out some limitations with regard to the usage of such measures. They indicated that one of the most important factors impairing SI or AI is fluctuating noise, especially speech embedded in fluctuating maskers, e.g., competing talkers [3,23,24]. Lombard effect is another factor impeding SI or speech quality evaluation [25]. More limitation factors were identified in the context of hearing aids, i.e., peak-clipping and center-clipping distortions in the speech signal [26], cochlear implants, i.e., STOI (Short-Time Objective Intelligibility) [27], or noise suppression algorithms [28]. The thread related to creating SI-based metrics derived from the time-domain approaches such as Envelope Regression (ER) was found to be promising in acoustically degraded environments with multiple talkers and speaking styles [4]. ER is a time-domain STI method that works as a function of the window length [4]. Moreover, Payton and Shrestha [4] concluded that short-term windows might be more appropriate than a long-term analysis so that distortions during gain transitions do not necessarily distort predicted intelligibility during steady-state intervals.

While discussing speech intelligibility, approaches to speech quality assessment based on perceptual principles should also be brought [29]. Among them, Perceptual Evaluation of Speech Quality (PESQ) [30,31], designed by ITU-T P.862 [32], utilized for narrowband speech with minor impairments, should be mentioned [33]. Another method, i.e., ITU-T P.563 [34], allows for dealing with narrowband speech quality. 

Moreover, in recent years, one can see a new way to deal with speech assessment based on machine learning [35,36,37]. Most of these approaches are designed for automatic speech quality evaluation. However, it may be assumed that the same paradigm will more often be used for speech intelligibility assessment based on deep learning in the future [35,38].

### 1.2. STI as an Objective Speech Intelligibility Measure

Before the first attempts were made to use objective measures of intelligibility, subjective methods were used, demanding the involvement of many individuals in the process and thorough statistical analysis. The choice of the test material was also a problem. One of the first attempts to formalize subjective tests of speech intelligibility was an IEEE recommendation published in 1969 [39]. It defines three types of tests (Isopreference, Relative Preference, and Category-Judgment), describes speakers, listeners, and speech loudness requirements, and provides a list of phonetically balanced sentences (in English) that could serve as test material. Even modern researcher study uses this list (e.g., [40]). However, a literature review reveals a wide variety of subjective tests, mainly in the selection of test material. Often nonsense words or syllables are used [40,41,42]. This group also includes the so-called logatoms-short pseudo-words [43,44]. In the case of sentences, the test material is usually constructed to make it difficult to predict the successive words. Such sentences are grammatically correct but do not carry meaningful, semantic information. Their length is limited to a few words. This type of list is described, for example, by Lavandier [45] or Ozimek [46]. Some studies are based on several tests—containing words and sentences—to investigate speech intelligibility in diverse conditions (e.g., [47]).

Subjective tests are definitely more time-consuming than objective tests, and the results obtained are difficult to compare with one another, for example, due to the different participants. However, this last disadvantage may prove to be an advantage in some situations. The selection of an appropriate group of participants provides an opportunity to assess the intelligibility of speech, e.g., for people with hearing impairments. 

Subjective tests are also capable of considering features typical for a given language. Unfortunately, some objective measures, such as, e.g., the STI, will provide inconsistent results in this situation. The studies of Kitapci and Galbrun ([47,48]) are a good example—the results obtained show that changes in STI values affect intelligibility differently depending on a particular language. 

It can be said that this approach was intentional in the design of the STI—it primarily enables evaluation of the transmission channel quality, leaving aside issues related to speakers or listeners. In contrast to the STI, the speech intelligibility index (SII) enables accounting for listeners’ hearing impairments, e.g., by including a subject’s audiogram in the calculations. In a simplified way, it is also possible to simulate specific frequency dependencies existing in a given speech or language [49]. The disadvantage of SII is an inability to consider, e.g., reverberation—the method focuses on assessing the effect of stationary noise on speech intelligibility. Rhebergen et al. in [50] proposed an extension of SII that takes into account the presence of nonstationary noise (called ESII), and George et al. [49] attempted to combine the properties of SII and STI.

Binaural hearing is another factor that should be considered in this type of analysis. Binaural hearing improves speech intelligibility in complex environments (e.g., reverberant conditions). Typically, single microphones with omnidirectional characteristics are used for STI measurements. As a result, the results may not be as good as those obtained with human participants. However, research is underway on a version of STI that would consider the binaural properties of human hearing (e.g., [51]).

Other noteworthy STI measure modifications are its simplifications—whose examples among many are STIPA and RASTI. STIPA (STI for public address systems) is a simplified version of the STI measure (e.g., using only seven modulation frequencies instead of the original 14 frequencies) designed to be used in portable speech intelligibility measurement devices [52]. RASTI (rapid speech transmission index) is computed based on only two-octave bands (500 Hz and 2 kHz), which makes the method faster to perform, but sometimes too inaccurate for some more complicated condition uses. Therefore, it was mainly employed for signals limited to the telephone acoustic frequency band [53].

### 1.3. Limitations of STI as a Way of Assessing Speech Intelligibility

STI is an objective measure that correlates with the degree of intelligibility of human speech at a point in space where it was measured. One of the assumptions of the measurement methodology is the use of a known excitation signal in the form of a noise signal being the sum of narrowband noise signals modulated by pair of modulating frequencies. The center frequencies of the carrier bands and the modulating frequencies are constant with time, and their values are defined in BS EN 60268-16. The algorithm for calculating the STI measure assumes that the component frequencies of the measurement signal do not change during the measurement. This fact makes the STI measure unsuitable for assessing the effect of nonlinear transformations applied in the audio path on speech intelligibility. Examples of this type of transformation are slowing down speech or compression of the dynamics of a speech signal. These transformations can affect the modulation frequency present in the STI-PA signal. Such a detuning causes the algorithm that computes the STI to have a greater modulation loss than it actually is. This is due to shifting the energy of modulation components from frequencies taken into account by the algorithm calculating STI to frequency ranges that this algorithm does not take into account. The STI measure enables the analysis of:

1. The impact of linear transformations on the intelligibility of a speech signal (e.g., filtration, adaptive filtering, signal amplification),
2. The effect of the appearance of additive disturbances (the disturbances being signals containing modulation signals, e.g., a real speech signal, are excluded).

For this reason, it is not possible to study the effect of the slowing down on speech intelligibility using the STI signal because it is a nonlinear transformation that changes the frequency structure of the signal, e.g., by changing the frequency of the carrier signals in the excitation signal. Therefore, to obtain a measure to objectively analyze the effect of the slowdown of the speech signal on speech intelligibility, the methodology for computing the STI has to be modified. This way the nature of the slowdown transformation can be taken into account in the analysis.

Possible extensions could envision applying such an approach to TIPA and STIPA and other MTF-based methods if they fail when dealing with the nonlinear enhancement of speech intelligibility.

## 2. Methods

As already said, we propose a modification of the standard STI method, which makes it possible to use with standard STI signals altered by the reverberant environment or a DSP algorithm in a nonlinear manner. To assess if our results are reliable, we performed a repeatability analysis and measured correlation with the baseline STI values associated with impulse responses used for tests.

### 2.1. Calculation of the Broadband STI Measure (bSTI)

The analysis of the amount of modulation transmitted by the public address system with the bMTF parameter is complicated due to interpretation problems. This is caused by the fact that the standard metric for measuring speech intelligibility is not the amount of modulation transmitted by the channel (MTF) but the STI measure, which is derived from the value calculated based on the MTF amount. In this paper, we propose a method utilizing the MTF concept along with perceptual corrections derived from the STI standard. This evolves into a bSTI measure, which may be applied to situations where nonlinearly processed speech quality should be assessed.

The definition of the STI measure is based on the concept of the modulation transfer function (MTF). In determining the MTF for calculating the STI, a measure of the amount of modulation contained in the analyzed signal (denoted by MD) is used. For this purpose, the correlation with orthogonal, sinusoidal reference signals is used according to the following formula:

(1) $$\mathrm{MD}=2\;\frac{\sqrt{{\left[\sum^{\;}I_{k}\left(t\right)\sin\left(2\pi f_{m}t\right)\right]}^{2}+{\left[\sum \;I_{k}\left(t\right)\cos\left(2\pi f_{m}t\right)\right]}^{2}}}{\sum \;I_{k}\left(t\right)},$$

where t represents time, $I_{k}\left(t\right)$ is the acoustic intensity at the moment t for the *k*-th frequency band of the STI, and $f_{m}$ is a central frequency of the *k*-th frequency band.

The modulation content factor can be calculated both for the signal on the transmitting side (before processing in the public address system and propagation in the transmission channel) and on the receiving side (listener or measuring microphone). These factors are denoted as MDT and MDR, respectively. MTF is defined in this case by the following formula:

(2) $${\mathrm{MTF}}_{k,f_{m}}=\frac{{\mathrm{MDR}}_{k,f_{m}}}{{\mathrm{MDT}}_{k,f_{m}}},$$

The classical method of calculating MDR and MDT measures cannot be used for the signals for which the deceleration was used. For this reason, they have been modified and generalized to the entire acoustic band (not only to bands selected by index *k*):

(3) $${\mathrm{BMD}}_{k}=\frac{\sum_{n=1}^{N}\left|\mathrm{FFT}\left(I_{k}\left(t\right),n\right)\right|}{\sum \;I_{k}\left(t\right)},$$

where FFT stands for fast Fourier transform. *N* is the transform length, and *n* is the transform coefficient index. Similar to the measurement of the STI signal, we can distinguish the bMDR coefficient for the signal analyzed on the receiving side and bMDT for the signal analyzed on the transmitting side before entering the transmission channel. An additional change is that the bMTF measurement uses a signal composed of actual warning messages to be delivered in a tested space or other signals containing real human speech. The bMTF measure is calculated analogously to the MTF measure:

(4) $${\mathrm{bMTF}}_{k}=\frac{{\mathrm{bMDR}}_{k}}{{\mathrm{bMDT}}_{k}}.$$

**Figure 1:**
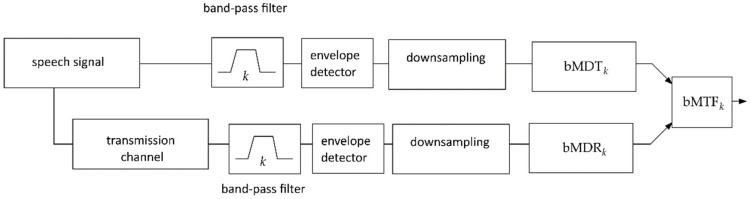
Flow chart showing computing broadband modulation transition factor over a transmission channel (bMTF).

The flow chart of the method of calculating the bMTF value is schematically presented in Figure 1. 

### 2.2. Practical Measurement of bSTI and Its Relation to STI

One of the difficulties connected to the measurement of bSTI is the choice of the excitation signal. As the definition of the bSTI does not assume that the system-under-test processes the signal only in a linear manner, it is also necessary to take that the output spectrum may contain additional frequency components, which are nonexistent in an original excitation signal. This imposes another challenge associated with the choice of the input signal because its structure may be altered in a more profound way than in linear systems. 

To overcome this issue, we decided to choose a measurement signal linked as closely as possible to speech intelligibility, which is most often the true metric optimized in the process of acoustic sound treatment. Thus, the signal we employed is a human speech in a language intended to be used the most in the place of measurement. In our case, we focused on signals generated from utterances spoken in the Polish language. The advantage of this approach is that even speech processed in a nonlinear manner may still be intelligible. There are examples in the literature provided, for instance, for a binarized speech signal, which was still comprehensible for listeners [54]. By binarization, we mean thresholding of a speech signal, similar to a very hard distortion that effectively causes the speech signal to have only two values—a “high one” and a “low one.” Therefore, we can rely on a human speech-based excitation signal to measure the intelligibility of even nonlinearly processed speech. Such a measurement can be possibly carried out even in the case of such a destructive operation as speech binarization. 

The choice of the type of measurement signal is just an initial step to designing an excitation signal. It is also necessary to determine the length of such a signal. On the one hand, it should be long enough to provide accurate and repeatable results. On the other hand, it should be reasonably short to keep the length of measurements in a reasonable range. This is because lengthy measurements, changing acoustic conditions inside a room, e.g., due to changing street traffic noise in a nonideally insulated space, may impose a challenge. 

**Figure 2:**
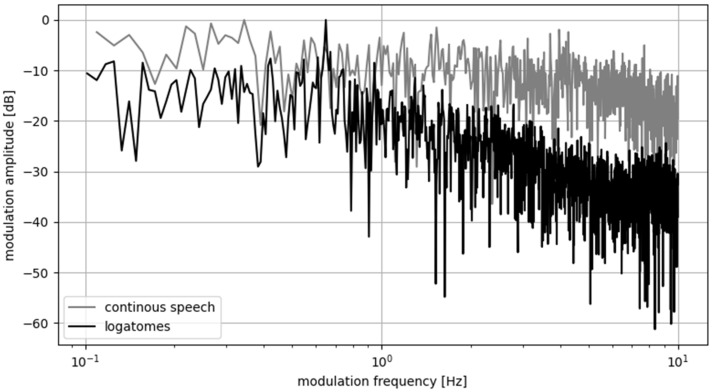
Modulation frequency spectra of the signal built from logatomes (the black curve) and a signal generated from the continuous speech (the gray curve). A harmonic component with a frequency of 0.67 Hz is visible for the logatome-based signal, which corresponds to the speech rate of 40 words per second. Modulation envelopes were calculated for the carrier frequency of 1000 Hz.

Another problem is the structure of the excitation signal. In the initial experiment, we used isolated words as components of the excitation signal. Words were repeated in a signal with a constant rate (measured in words per second). However, we found that this caused a serious problem because such a repeatable structure of excitation signal introduced an unwanted envelope modulation that was hard to remove from the measured signals. For instance, for a speech rate of 40 words per minute (0.67 words per second), additional frequency components of 0.67 Hz and its harmonics are introduced. The resulting modulation spectrum is shown in Figure 2. This unwanted frequency component also influenced the measurement of the bSTI metric to the degree that we decided to change the excitation signal structure. 

Consequently, the choice was a composite signal consisting of utterances concatenated into a longer signal. Due to such a structure, the signal consists primarily of continuous speech. Utterances are concatenated on fragments containing only silence. Thus, it was possible to significantly reduce the amount of unwanted structural modulation frequencies in an envelope of the excitation signal. The excitation signal structured this way provided results of bSTI that correlate better with the STI ground truth measurements than when a signal is generated by concatenation of single word recordings uttered in isolation and not being a part of a longer, sentence-long utterance.

## 3. Results

### 3.1. Results for Excitation Signal Synthesized from Polish Utterances

Assessment of applicability of the bSTI measure was based on STI measurements performed in 10 locations of 3 interiors, marked as A, B, C. Spa A is a large volume shopping mall, is high, and has several levels. Its total cubature is 130,000 cubic meters. A single floor has an area of corridors and passages of approximately 2300 square meters. Interior B is a garage of the shopping mall. It is relatively low and vast, with a total area of 12,000 square meters and a height of 2.5 m. Interior C is a staircase. This interior consists of a communication route and two-flight stairs. Wall surfaces are putty and finished with acrylic paint. The floor and stairs are finished with ceramic tiles. The cubature of interior C is 95 m^3^. The multitude of hard reflecting surfaces is conducive to a high reverberation. In all spaces, public address systems were employed to measure impulse responses using the correlation technique. Detailed characteristics of the reverberation time (for octave bands with center frequencies: 250, 500, 1000, 2000, 4000, 8000 Hz, and average value) for each impulse response are presented in Table 1. Reverberation time was calculated using the Dirac ver. 4 Bruel & Kjaer software. STIPA indicator was measured in the direct way using NTI AL1 Analyzer.

**Figure 3:**
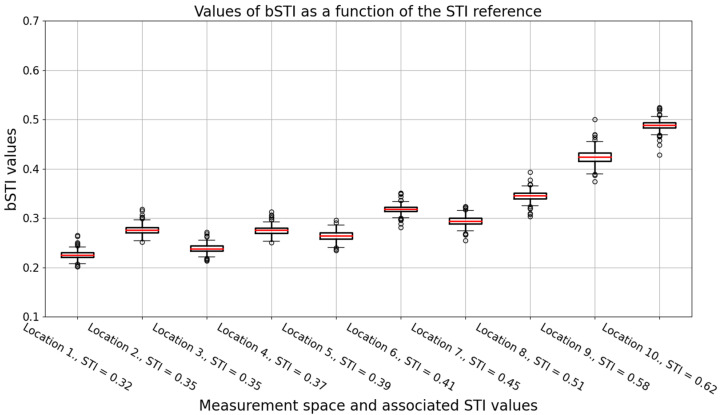
A boxplot visualizing the bSTI measures obtained for each impulse response. Data acquired from all excitation signal lengths were used; thus, each box represents a result of the 200 bSTI-based calculations. Results are obtained for utterances spoken in Polish. Medians of each data series are denoted with red lines, circles denote outlier points.

Impulse responses of each location were used to calculate the bSTI measure, starting with convolving a bSTI measurement signal and the excitation and then processing the result of such convolution to obtain the bSTI value. The problem of such a calculation is the choice of a measurement signal length. We decided to test the duration from 30 s up to 300 s with a 30 s increment, which resulted in 10 possible sizes of the excitation signal. Each excitation signal was tested with 20 randomly generated recordings obtained by concatenating Polish native speakers utterances of 4 speakers (2 males and 2 females). Speech signals in such excitation signals did not entirely fill in the desired durations, but the average error of the algorithms was 92 ms, so it was suitable for the measurement of bSTI. It should be noted that speech signal by itself contains much more near-silent moments than the aforementioned 92 ms, which were padded with silence. Such a calculation made it possible to calculate the bSTI measure for each excitation signal processed by each of the 10 impulse responses associated with varying values of STI. A boxplot visualizing the relation between the bSTI measure and the STI is depicted in Figure 3.

It can be observed that the relation in Figure 3 seems not to be linear. However, it is not a consequence of the nonlinear relationship between the STI and bSTI measurements, but the values of STI measured in 10 selected locations in which impulse responses were analyzed in the study. The degree of a linear relationship between STI and bSTI measurements can be assessed by calculating Pearson’s correlation coefficient. Such an analysis will be carried out later on in this section.

**Figure 4:**
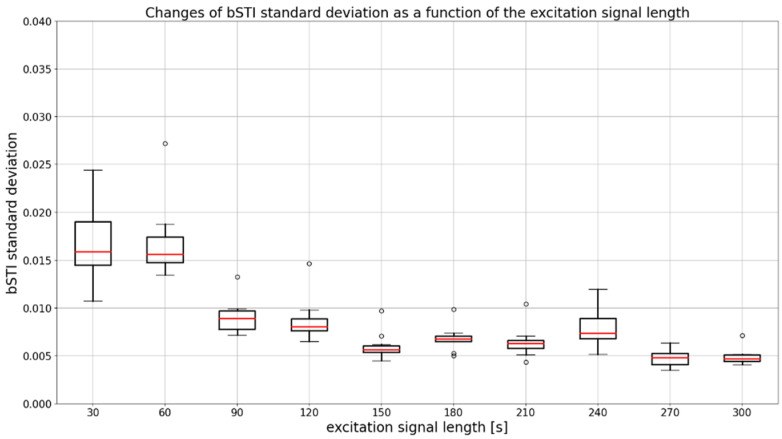
A boxplot visualizing a standard deviation of bSTI measurement for each excitation signal duration. Results are obtained for utterances spoken in Polish. Medians of each data series are denoted with red lines, circles denote outlier points.

An important factor in measuring the bSTI measure is the choice of the measurement signal duration. Therefore, we decided to investigate how the choice of an excitation signal duration affects the standard deviation of obtained bSTI measurements. As the calculation of bSTI was repeated 20 times for each excitation signal duration, it was possible to prepare a dataset visualized in Figure 3 and Figure 4. Each bSTI value obtained in this process results from a different, random excitation signal consisting of Polish utterances. As there are 10 locations investigated in such a way, the total number of bSTI values calculated in this process is 2000. A standard deviation is derived based on the results of bSTI values obtained for each of the 10 sites.

As can be seen in Figure 4, the standard deviation decreases for excitation signal durations above 120 s. Such a hypothesis can further be tested by employing statistical methods such as ANOVA and Kruskal–Wallis test. For all statistical tests presented in this study, a significance level of 0.05 was assumed. Tests were carried out with the use of Python programming language. A SciPy (version 1.7.3) and Statsmodels (version 0.13.1) programming libraries were used in this process. In the case of data from Figure 4, ANOVA cannot be applied because variances of all measurement groups (related to the excitation signal duration) are not equal. This fact was confirmed with the use of the Levene statistical test, which yielded a test statistic of 2.13, resulting in a *p*-value lesser than 0.03. Based on that, it may be concluded that a non-parametric ANOVA alternative in the form of a Kruskal–Wallis test has to be employed. The statistic of the aforementioned test results in a value of 78.41, which leads to a *p*-value lesser than 0.001. Thus, one has to conclude that at least one of the differences observable in Figure 4 is statistically significant. Therefore, Dunn’s post hoc test can be carried out to find out which results (for which pairs of durations) are different in a statistically significant way. A matrix of *p*-values of Dunn’s post hoc test is shown in Table 2. 

**Figure 5:**
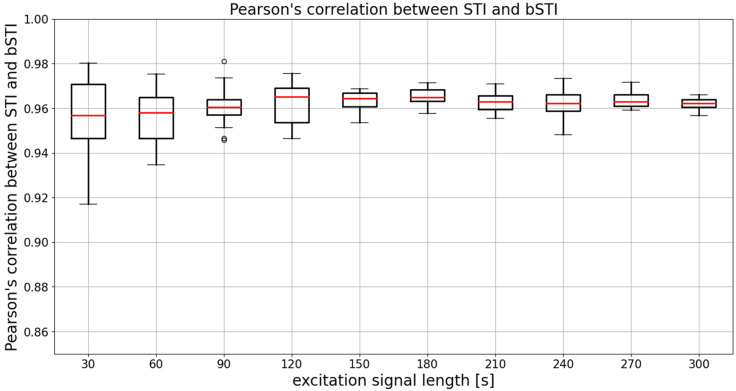
Results of Pearson’s correlation coefficient calculated for each considered excitation signal duration. Results are obtained for utterances spoken in Polish. Medians of each data series are denoted with red lines, circles denote outlier points.

Another way of evaluating the bSTI measurement method is the evaluation of the linear relationship between the STI measurement (performed on-site, during the impulse response acquisition) and the series of bSTI values calculated with the use of 20 randomly generated excitation signals (20 signals for each duration). Pearson’s correlation coefficient can be calculated for the reference STI values of 10 impulse responses and associated bSTI values. This calculation can be repeated 20 times as each random bSTI measurement was also generated 20 times. Results of such analyses are shown in Figure 5.

**Figure 6:**
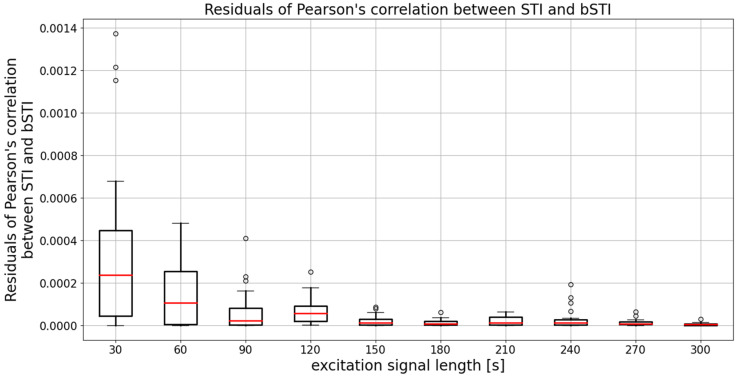
A boxplot showing values of residuals calculated for each of the considered excitation value durations. Results are obtained for utterances spoken in Polish. Medians of each data series are denoted with red lines, circles denote outlier points.

The mean (averaged across all excitation signal durations) median Pearson’s correlation factor is 0.961. This means that the degree of correlation between STI and bSTI is high, and bSTI can be a good measure derived from the STI and can be used in similar situations as the STI. However, as can be seen in Figure 5, the choice of the excitation signal duration determines the level of variance of obtained results. The median is similar in each case, and this fact is also confirmed by statistical analysis. The Levene test in the case of data from Figure 5 yielded the test statistic of 11.02, which results in a *p*-value lesser than 0.001. This means that variances of the data are not equal. Next, the Kruskal–Wallis test resulted in a test statistic of 14.61, which results in a *p*-value of 0.10. This leads to the conclusion that there are no statistically significant differences between medians of data presented in Figure 5. Therefore, the initial observation that the main difference between groups is defined by variance and not the medians is correct. To further test the variances of data from Figure 5, residuals of Pearson’s correlation coefficient were calculated in the following manner:

(5) $$\rho ={\left(r\left(d\right)-\bar{r}\left(d\right)\right)}^{2},$$

where ρ is a vector of residual values calculated for the vector r(d) containing all Pearson’s correlation coefficient values for the excitation signal length of d, and the $\bar{r}\left(d\right)$ is a mean value of r(d). A boxplot showing the residual values ρ is depicted in Figure 6.

To prove the variance of two given groups, one can use means of medians of their respective residuals. Therefore, an ANOVA or Kruskal–Wallis test can be applied to data from Figure 6, and conclusions from such analysis can be used to find out which variances depicted in Figure 6 differ in a statistically significant way. Levene test of data from Figure 6 returned a test statistic of 13.47, and thus a Kruskal–Wallis test has to be employed for further analysis. The test statistic of such an examination is 49.25, which is associated with a *p*-value lesser than 0.001. Therefore, one can conclude that at least one pair of data sets from Figure 6 has medians that differ in a statistically significant way, and Dunn’s post hoc test can be carried out to find out such differences. Results of such an analysis are contained in Table 3. Grey-shaded areas indicate values that differ in a statistically significant way.

As shown in Table 3, the length of the excitation signal is a critical parameter for the reliability of the proposed method. The shortest viable length of the excitation signal is 150 s. There are no statistically significant differences between measurements performed with excitation signal lengths of 30, 60, and 90 s. Similarly, there are no differences for measurements carried out with signals having the duration of 270 s and 300 s. The longest-considered signals are a good choice if high precision and repeatability of measurement results are needed.

### 3.2. Results for Excitation Signal Synthesized from English Utterances

**Figure 7:**
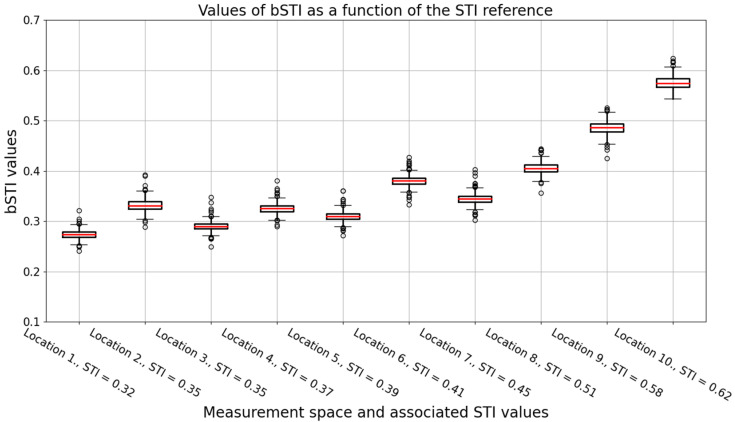
A boxplot visualizing the bSTI measures obtained for each impulse response. Data acquired from all excitation signal lengths were used; thus, each box represents a result of the 200 bSTI-based calculations. Results are obtained for utterances spoken in English. Medians of each data series are denoted with red lines, circles denote outlier points.

Similar to the experiment using Polish utterances, it is possible to perform an investigation based on excitation signals employing English utterances spoken by native speakers from the MODALITY corpus, available online [55]. The experiment was carried out in the same manner as based on Polish utterances. The only difference was the language. The resulting plot of STI as a function of bSTI is provided in Figure 7.

**Figure 8:**
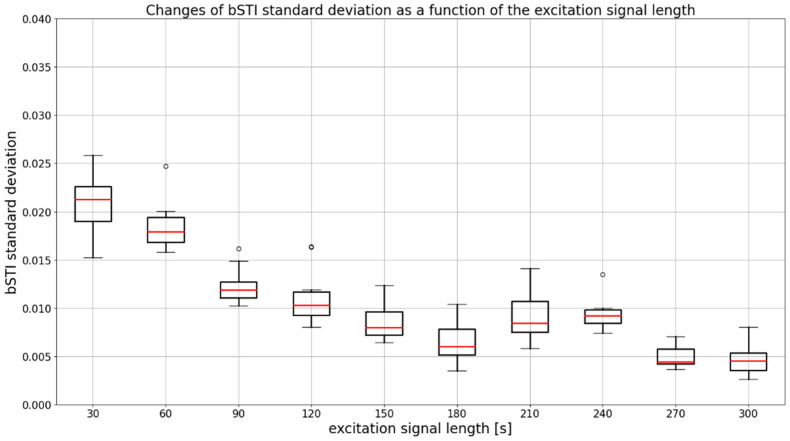
A boxplot visualizing a standard deviation of the bSTI measurement for each excitation signal duration. Results are obtained for utterances spoken in Polish. Medians of each data series are denoted with red lines, circles denote outlier points.

The result is similar to the case of the Polish language, however, values of the bSTI measures are higher than corresponding values derived from the Polish language utterances. The stability of the results obtained was also investigated, and the outcomes of such an analysis (analogous as in the case of the Polish language) are shown in Figure 8. 

In terms of stability of obtained results, the outcomes are similar as in the case of the Polish language, however, repeatability of results can be obtained for signals slightly longer than polish ones. For this case, signals should be at least 180 s long. 

To further investigate differences between signals of different lengths, statistical analysis was carried out. For differences of bSTI measures, a Kruskal–Wallis test was carried out (the same test was used for signals in Polish). The test statistic value was equal to 81.56, which results in *p*-value lesser than 0.001, and therefore, a Dunn’s post hoc test can be carried out. The outcome of the post hoc test is depicted in Table 4.

**Figure 9:**
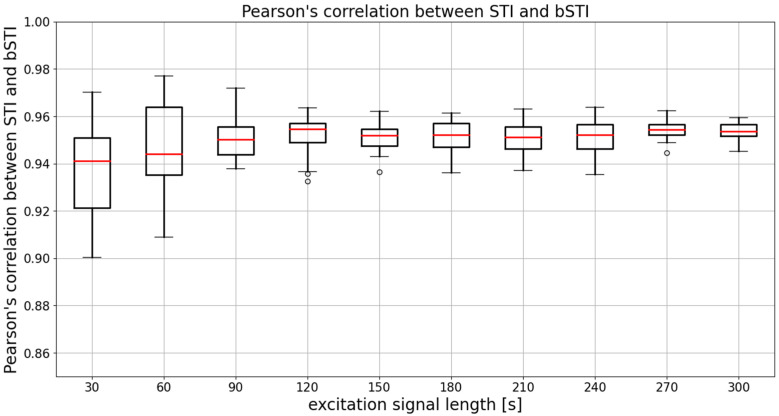
Results of Pearson’s correlation coefficient calculated for each considered excitation signal duration. Results are obtained for utterances spoken in English. Medians of each data series are denoted with red lines, circles denote outlier points.

**Figure 10:**
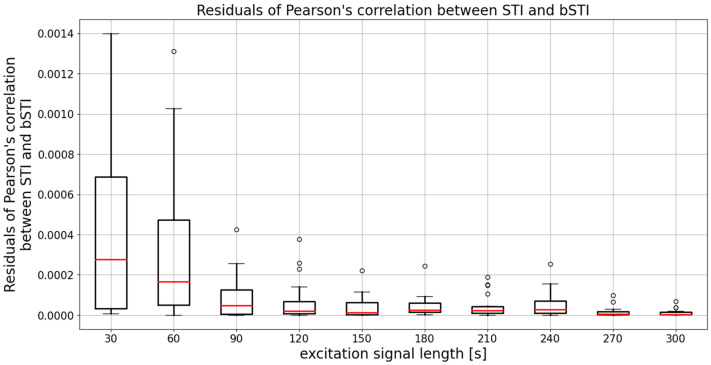
A boxplot showing values of residuals calculated for each of the considered excitation value durations. Results are obtained for utterances spoken in English. Medians of each data series are denoted with red lines, circles denote outlier points.

A boxplot analysis depicting acquired Pearson’s correlation coefficient for selected lengths of the excitation signals is shown in Figure 9. As can be seen, correlation values stabilize for excitation signals of duration equal to 90 s or longer. The variance of obtained Pearson’s correlation factors also decreases with the size of the excitation signals. It stabilizes for signals with a length of 90 s or longer, which can be seen in Figure 10. 

To find out if the decrease of variance is statistically significant, a test on residuals of correlation values has to be performed. First, a Kruskal–Wallis test was carried out and resulted in a test statistic of 17.81. Thus, the *p*-value of the test is equal to 0.04, and therefore a post hoc test can be carried out to find out which data groups from Figure 10 differ in a statistically significant manner. Results of this post hoc test are shown in Table 5.

Results of Dunn’s post hoc test lead to a conclusion that variance decrease observable for signals shorter than 90 s is statistically significant. In conclusion, excitation signals obtained from English language-based utterances also yielded results that are of quality, useful for estimation of speech intelligibility in a manner similar to the STI method. 

## 4. Conclusions

We found that the proposed bSTI measure is highly correlated with the STI measure. This relationship resulted in Pearson’s correlation factor of 0.961 in the case of the Polish language and 0.950 in the case of English. Thus, this measure can be used as a substitute for STI measure in cases where the classical STI is not applicable. An example of such a situation is the measurement of systems employing nonlinear speech intelligibility enhancement methods based on, e.g., artificially slowing down the speech signal in reverberant conditions [11]. For such a case, the classical STI measure is not applicable, while the bSTI measure is. 

The last observation is that repeatability of Pearson’s correlation coefficient value was also obtained for excitation signals having lengths of 150 s and larger. Therefore, the recommendation of using excitation signals no shorter than 150 is also confirmed by analyses employing Pearson’s correlation factor. This conclusion is valid for both Polish- and English-based bSTI excitation signals.

Our modification of the STI method has the potential to be a simple alternative to STI and STIPA measures if a measurement of a nonlinear system is needed. As it is an STI-derived measure, it is correlated to the STI in standard use cases, which was shown in the study. The advantage of bSTI is that it can also be employed for the measurement of systems for which the classical STI-based measurement is not applicable. Therefore, if someone is accustomed to the interpretation of STI-based measurements, understanding of bSTI-based ones would be analogous. Additionally, measurement with the use of human speech signals provides a potential for obtaining results that are more representative of the quality of the system-under-measurement as the language of utterances is also a factor influencing the outcome of a measurement. The properties of a given utterance language are not taken into account in the case of standard STI-based measurement. Thus, the bSTI-based measurement method may be useful if it is essential to know the differences between intelligibility of utterances in different languages.

In the future, we would like to extend the database of impulse responses that were used for testing the introduced measure. This would validate our method in real-life conditions, which does not simulate room by utilizing impulse response to process the bSTI measurement signal. Such an approach would allow verifying that practical examples of the bSTI measurement confirm the results shown in this manuscript. It should be noted that our current approach has to rely on the processing of the excitation signals with impulse responses. This way, it is possible to test thousands of room–excitation signal combinations necessary to estimate the reliability and repeatability of bSTI measurements.

## Figures and Tables

**Table 1:** RT of each location along with the STIPA values.

Location	Interior	RT 250	RT 500	RT 1000	RT 2000	RT 4000	RT 8000	RT AVG	STIPA
1	C	4.331	3.749	3.140	2.744	2.036	1.027	2.838	0.32
2	A	2.223	2.624	2.520	2.623	2.026	1.507	2.254	0.35
3	C	3.388	3.129	3.384	2.684	1.909	1.002	2.583	0.35
4	B	3.103	2.934	2.674	2.412	2.067	1.452	2.440	0.37
5	C	3.205	3.196	3.347	2.599	1.892	1.060	2.550	0.39
6	A	2.343	2.594	2.648	2.579	2.033	1.412	2.268	0.41
7	C	1.363	2.063	2.050	1.805	1.437	0.830	1.591	0.45
8	C	1.516	1.211	1.399	1.269	0.997	0.653	1.174	0.51
9	B	2.133	1.964	1.765	1.438	1.079	0.858	1.540	0.58
10	A	2.013	2.358	2.372	2.439	1.676	1.277	2.023	0.62

**Table 2:** Dunn’s post hoc table for differences between bSTI values obtained for the Polish language-based excitation signals of different durations. Grey-shaded areas indicate values that differ in a statistically significant way.

Duration [s]	30	60	90	120	150	180	210	240	270	300
30	–	0.975	0.065	0.034	<$10^{-3}$	<$10^{-3}$	<$10^{-3}$	0.004	<$10^{-3}$	<$10^{-3}$
60	0.975	–	0.070	0.037	<$10^{-3}$	<$10^{-3}$	<$10^{-3}$	0.005	<$10^{-3}$	<$10^{-3}$
90	0.065	0.070	–	0.781	0.007	0.074	0.021	0.316	<$10^{-3}$	<$10^{-3}$
120	0.034	0.037	0.781	–	0.015	0.131	0.042	0.469	<$10^{-3}$	<$10^{-3}$
150	<$10^{-3}$	<$10^{-3}$	0.007	0.015	–	0.359	0.694	0.089	0.215	0.241
180	<$10^{-3}$	<$10^{-3}$	0.074	0.131	0.359	–	0.600	0.432	0.031	0.037
210	<$10^{-3}$	<$10^{-3}$	0.021	0.042	0.694	0.600	–	0.190	0.102	0.118
240	0.004	0.005	0.316	0.469	0.089	0.432	0.190	–	0.003	0.004
270	<$10^{-3}$	<$10^{-3}$	<$10^{-3}$	<$10^{-3}$	0.215	0.031	0.102	0.003	–	0.945
300	<$10^{-3}$	<$10^{-3}$	<$10^{-3}$	<$10^{-3}$	0.241	0.037	0.118	0.004	0.945	–

**Table 3:** Dunn’s post hoc table for residual values calculated from Pearson’s correlation factors obtained for the Polish language-based excitation signal. Grey-shaded areas indicate values that differ in a statistically significant way.

Duration [s]	30	60	90	120	150	180	210	240	270	300
30	–	0.233	0.004	0.237	<$10^{-3}$	<$10^{-3}$	<$10^{-3}$	0.001	<$10^{-3}$	<$10^{-3}$
60	0.233	–	0.090	0.991	0.022	0.002	0.018	0.043	0.004	<$10^{-3}$
90	0.004	0.090	–	0.088	0.546	0.148	0.507	0.741	0.221	0.014
120	0.237	0.991	0.088	–	0.021	0.002	0.018	0.042	0.003	<$10^{-3}$
150	<$10^{-3}$	0.022	0.546	0.021	–	0.400	0.952	0.785	0.535	0.064
180	<$10^{-3}$	0.002	0.148	0.002	0.400	–	0.435	0.265	0.825	0.312
210	<$10^{-3}$	0.018	0.507	0.018	0.952	0.435	–	0.739	0.575	0.073
240	0.001	0.043	0.741	0.042	0.785	0.265	0.739	–	0.372	0.034
270	<$10^{-3}$	0.004	0.221	0.003	0.535	0.825	0.575	0.372	–	0.218
300	<$10^{-3}$	<$10^{-3}$	0.014	<$10^{-3}$	0.064	0.312	0.073	0.034	0.218	–

**Table 4:** Dunn’s post hoc table for differences between bSTI values obtained for the English language excitation signals of different duration. Grey-shaded areas indicate values that differ in a statistically significant way.

Duration [s]	30	60	90	120	150	180	210	240	270	300
30	–	0.683	0.074	0.014	<$10^{-3}$	<$10^{-3}$	<$10^{-3}$	0.001	<$10^{-3}$	<$10^{-3}$
60	0.683	–	0.168	0.040	0.001	<$10^{-3}$	0.002	0.003	<$10^{-3}$	<$10^{-3}$
90	0.074	0.168	–	0.503	0.043	0.001	0.080	0.118	<$10^{-3}$	<$10^{-3}$
120	0.014	0.040	0.503	–	0.177	0.009	0.281	0.371	<$10^{-3}$	<$10^{-3}$
150	<$10^{-3}$	0.001	0.043	0.177	–	0.201	0.787	0.649	0.028	0.023
180	<$10^{-3}$	<$10^{-3}$	0.001	0.009	0.201	–	0.121	0.083	0.359	0.320
210	<$10^{-3}$	0.002	0.080	0.281	0.787	0.121	–	0.853	0.014	0.011
240	0.001	0.003	0.118	0.371	0.649	0.083	0.853	–	0.008	0.006
270	<$10^{-3}$	<$10^{-3}$	<$10^{-3}$	<$10^{-3}$	0.028	0.359	0.014	0.008	–	0.939
300	<$10^{-3}$	<$10^{-3}$	<$10^{-3}$	<$10^{-3}$	0.023	0.320	0.011	0.006	0.939	–

**Table 5:** Dunn’s post hoc table for residual values calculated Pearson’s correlation factors obtained for the English language-based excitation signal. Grey-shaded areas indicate values that differ in a statistically significant way.

Duration [s]	30	60	90	120	150	180	210	240	270	300
30	–	0.650	0.025	0.006	<$10^{-3}$	0.012	0.002	0.010	<$10^{-3}$	<$10^{-3}$
60	0.650	–	0.074	0.021	0.001	0.039	0.007	0.032	<$10^{-3}$	<$10^{-3}$
90	0.025	0.074	–	0.602	0.112	0.781	0.352	0.722	0.005	0.005
120	0.006	0.021	0.602	–	0.285	0.808	0.682	0.868	0.024	0.022
150	<$10^{-3}$	0.001	0.112	0.285	–	0.190	0.510	0.217	0.233	0.224
180	0.012	0.039	0.781	0.808	0.190	–	0.514	0.939	0.012	0.012
210	0.002	0.007	0.352	0.682	0.510	0.514	–	0.564	0.064	0.061
240	0.010	0.032	0.722	0.868	0.217	0.939	0.564	–	0.015	0.014
270	<$10^{-3}$	<$10^{-3}$	0.005	0.024	0.233	0.012	0.064	0.015	–	0.983
300	<$10^{-3}$	<$10^{-3}$	0.005	0.022	0.224	0.012	0.061	0.014	0.983	–

## Data Availability

Not applicable.
